# Primary Angiosarcoma of the Urinary Bladder Without Prior Radiation Exposure: A Case Report

**DOI:** 10.1002/iju5.70309

**Published:** 2026-11-13

**Authors:** Sho Nonoyama, Kyoko Koike, Keita Sekine, Yuichi Katsuoka, Ryosuke Hashimoto, Hirohito Kobayashi, Tomotaka Hattori

**Affiliations:** ^1^ Department of Urology Ebina General Hospital Ebina Kanagawa Japan; ^2^ Department of Pathology Ebina General Hospital Ebina Kanagawa Japan

**Keywords:** anemia, angiosarcoma, hematuria

## Abstract

**Introduction:**

Primary angiosarcoma of the urinary bladder is an exceptionally rare and aggressive endothelial malignancy, with fewer than 40 cases reported in the English‐language literature, making diagnosis challenging.

**Case Presentation:**

A 76‐year‐old woman presented with a 1‐week history of gross hematuria and severe anemia. Computed tomography demonstrated intravesical lesions without another bleeding source. Transurethral electrocoagulation and resection were performed despite poor visualization due to hematuria and blood clots. Histopathological examination showed a highly cellular neoplasm with predominantly epithelioid morphology, arranged in sheets and irregular vascular channel‐like structures. Immunohistochemistry revealed CD31 positivity and negativity for D2‐40, CD34, AE1/AE3, p63, and GATA3, supporting a diagnosis of angiosarcoma. Hematuria recurred postoperatively, and the patient declined further treatment because of her religious beliefs.

**Conclusion:**

Primary bladder angiosarcoma should be considered as a rare differential diagnosis in patients presenting with severe hematuria, particularly when hemostasis is difficult to achieve because of the tumor's highly vascular nature.

## Keynote Message

Primary bladder angiosarcoma should be considered in patients with severe hematuria, even without prior radiation exposure. Early histopathological diagnosis is important given its aggressive clinical course.

## 1 Introduction

Angiosarcoma is an uncommon, highly aggressive malignant neoplasm of endothelial origin that can arise in various organs. Primary involvement of the urinary bladder is exceedingly rare, with fewer than 40 cases reported in the English‐language literature (Table 1) [1, 2, 3, 4, 5, 6, 7, 8, 9, 10, 11, 12, 13, 14]. It predominantly affects older individuals, often with a history of pelvic irradiation, and typically presents with hematuria. Histologically, bladder angiosarcoma exhibits irregular vascular channels lined by atypical endothelial cells, although solid, spindle‐cell, and epithelioid patterns may also occur.

**TABLE 1:** Reported cases of radiation‐associated bladder sarcoma: Presentation, imaging, treatment, and prior radiation exposure.

Study	Symptoms	Imaging	Treatment	Prior radiation exposure
Bahouth 2015	Hematuria	CT/cystoscopy	TURBT + palliative RT	Yes, prostate
Cito 2021	Incidental mass	CT/CT urography	TURBT → Cystectomy	Yes, prostate
Elatreisy 2023	Severe gross hematuria + clot retention	CT	Embolization → Cystectomy + chemo	No
Kulaga 2007	Hematuria	NR	TURBT	Yes, endometrial
Matoso 2015	Hematuria + mass	NR	TURBT ± cystectomy	6/9 yes
Morgan 1989	Vaginal bleeding + hematuria	NR	Doxorubicin	Yes
Navon 1997	Hematuria	NR	Cystectomy	Yes, prostate
Ojerholm 2015	Hematuria	NR	Cystectomy	Yes, prostate
Rallabandi 2016	Hematuria	NR	TURBT	Yes, cervical
Seethala 2006	Hematuria	NR	Cystectomy + Gem/Doc	Yes, prostate
Tavora 2008	Hematuria ± irritative symptoms	NR	Biopsy/cystectomy	Some yes
Tynski 2017	Ascites + retention	CT	Palliative Gem/Doc	Yes, prostate
Wang 2016	Irritative LUTS + gross hematuria	Cystoscopy	TURBT → Cystectomy	Yes, prostate
Williams 2008	Hematuria	NR	TURBT → Cystectomy + chemo + RT	Yes, prostate

Abbreviations: Gem/Doc, gemcitabine/docetaxel; LUTS, lower urinary tract symptoms; NR, not reported; RT, radiation therapy; TURBT, transurethral resection of bladder tumor.

The rarity of bladder angiosarcoma and its variable histological appearance may lead to misdiagnosis, particularly when it is not considered in the differential diagnosis. Here, we report a case of primary urinary bladder angiosarcoma in a patient with no history of radiotherapy.

## 2 Case Presentation

A 76‐year‐old woman presented with a 1‐week history of gross hematuria, accompanied by dizziness but no lower urinary tract symptoms. Her medical history included hypertension, glaucoma, diabetes mellitus, left breast cancer, cerebral infarction, intracranial and aortic aneurysms, and schizophrenia. She had no history of radiotherapy.

On presentation, her vital signs were within normal limits. Physical examination revealed conjunctival pallor. Ultrasonography demonstrated an intravesical hyperechoic mass, suggestive of blood clot (Figure 1A). Laboratory data were notable for severe anemia (hemoglobin, 4.7 g/dL), hyponatremia (sodium, 120 mEq/L), and class II urine cytology. Computed tomography without contrast showed several intravesical tumors (Figure 1B) and a 10.8‐cm aortic aneurysm without endo‐leak. No apparent source of bleeding other than the tumors was identified. Furthermore, CT revealed neither another potential primary tumor site nor evidence of direct invasion from an adjacent tumor. MRI was not performed in this case because the patient was unable to follow commands or remain still during the examination.

**FIGURE 1:**
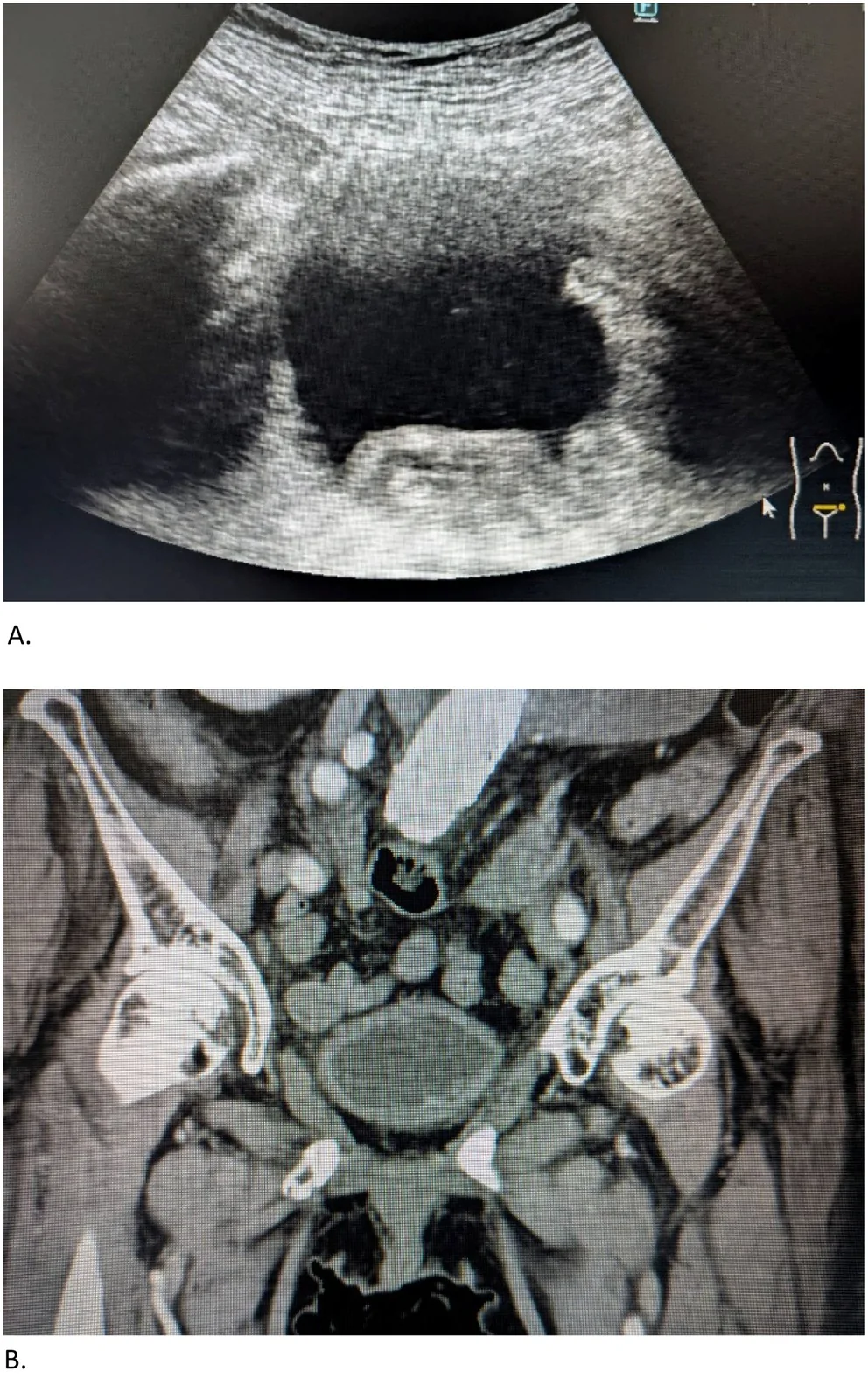
(A) Ultrasound image of the bladder. (B) CT images. Intravesical tumor on contrast CT.

A urinary catheter was placed, and blood transfusion was initiated upon admission. On hospital days 2 and 3, the hemoglobin level increased to 6.1 and 6.0 g/dL, respectively, but subsequently decreased to 4.9 g/dL on day 6. On hospital day 7, transurethral electrocoagulation and transurethral resection of a bladder tumor (TURBT) were performed. Intraoperatively, visualization was markedly impaired due to a large volume of intravesical clots. Following evacuation of the clots using an Ellik evacuator, erythematous and edematous mucosal changes with continuous oozing were identified at the trigone (Figure 2). The affected area was resected and sent for histopathological examination.

**FIGURE 2:**
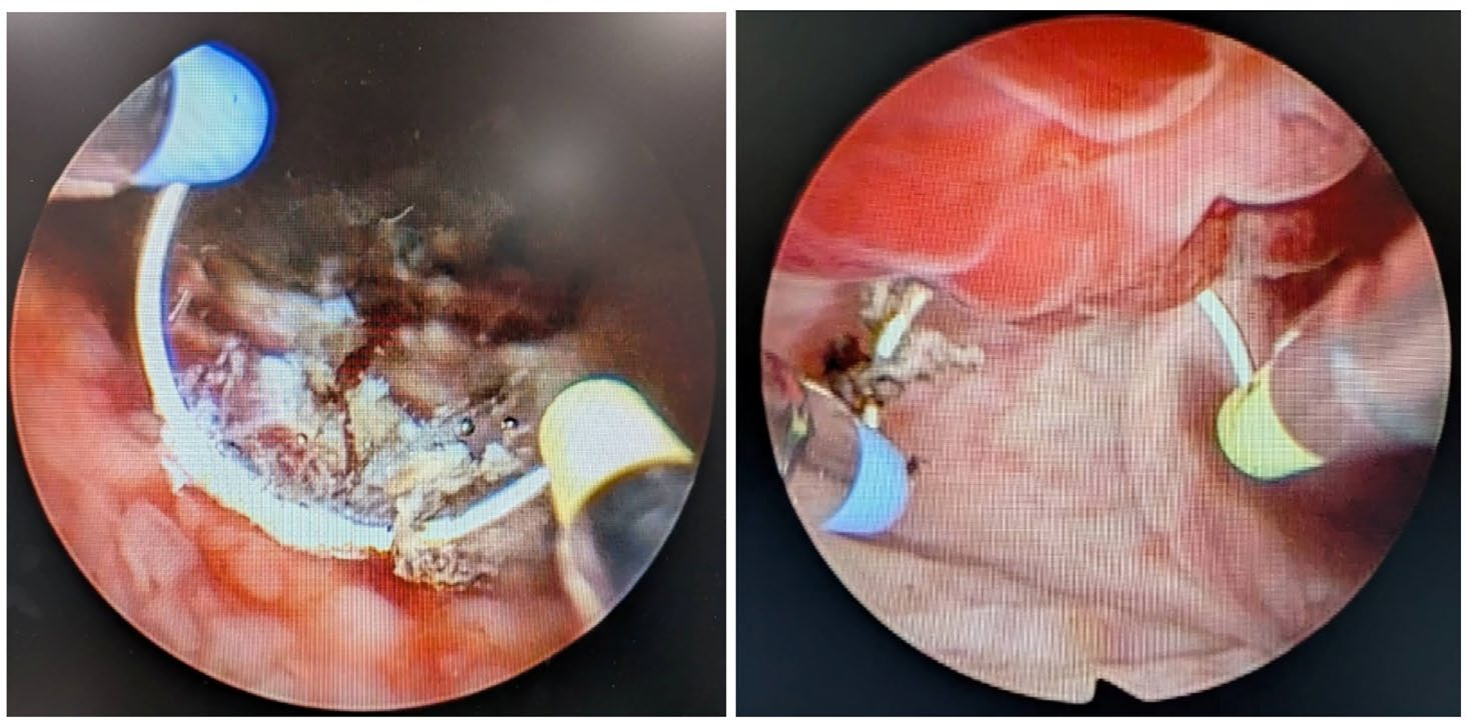
Intraoperative findings. The bladder mucosa demonstrated erythematous and edematous changes.

## 3 Pathology

### 3.1 Urinary Bladder (Transurethral Resection)

Histologically, the tumor diffusely infiltrated the lamina propria and was composed of a highly cellular neoplasm with predominantly epithelioid morphology, arranged in sheets and irregular vascular channel‐like structures. The neoplastic cells exhibited marked nuclear atypia with enlarged, hyperchromatic nuclei and prominent nucleoli (Figure 3A–C). The mitotic count was approximately 5–6 mitotic figures per HPF at ×400 magnification. Tumor necrosis was focal, involving approximately 10% of the tumor. Muscularis propria invasion could not be assessed because the muscularis propria was absent from the specimen. Immunohistochemically, the atypical proliferating cells were positive for CD31 and negative for D2‐40, CD34, AE1/AE3, p63, and GATA3 (Figure 3D–F). ERG immunohistochemistry was not performed. The Ki‐67 labeling index was approximately 70% (Figure 3G). The epithelioid morphology, expression of the endothelial marker CD31, and absence of epithelial markers supported a diagnosis of primary bladder angiosarcoma rather than metastatic breast carcinoma or urothelial carcinoma. Based on these histopathological and immunohistochemical findings, a diagnosis of primary bladder angiosarcoma was established. Although metastatic disease has been reported in the majority of patients at diagnosis, no evidence of metastasis was identified in our patient at presentation.

**FIGURE 3:**
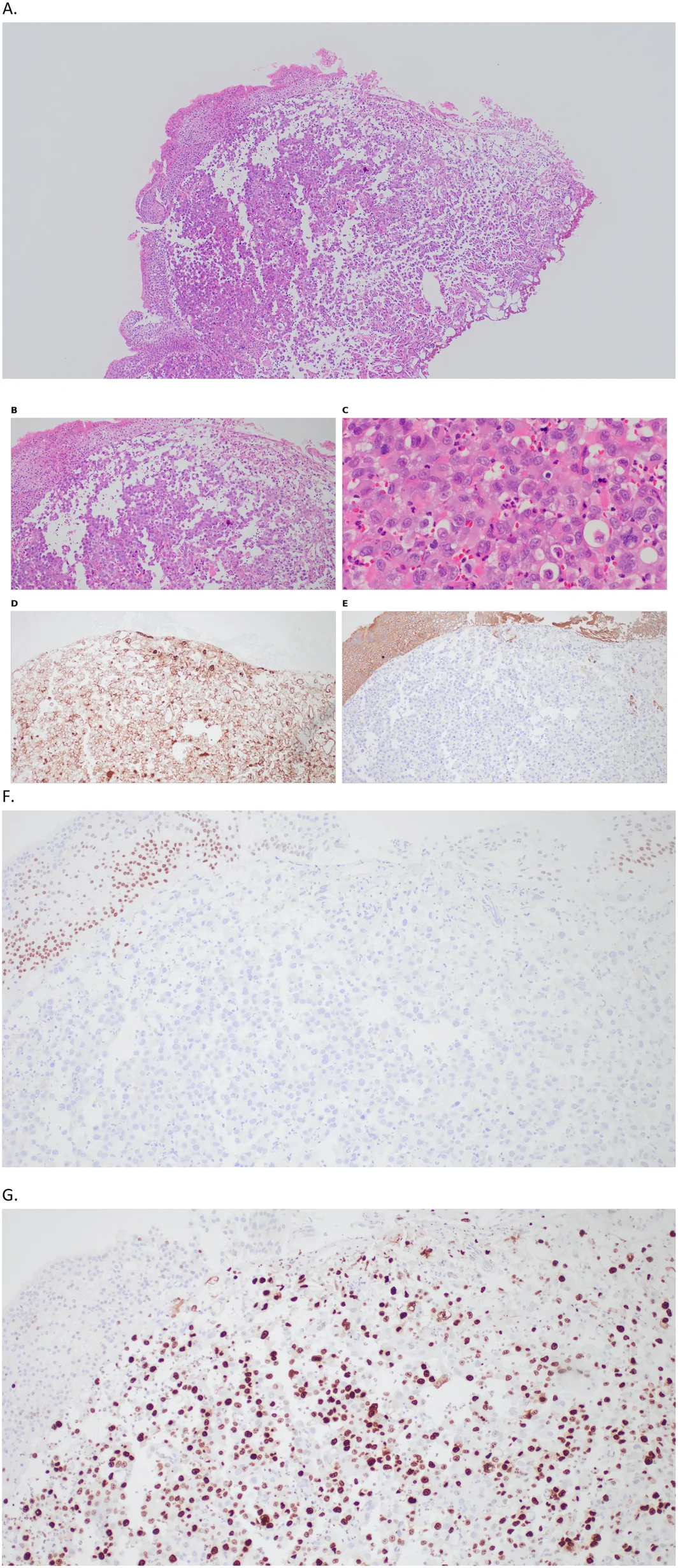
Histopathological and immunohistochemical features of the bladder angiosarcoma. (A–C) Hematoxylin and eosin (H&E) staining at ×40, ×100, and ×400 magnification, respectively. (D–G) Immunohistochemical staining for CD31, AE1/AE3, p63, and Ki‐67, respectively, at ×100 magnification. (A) HE×40. (B) H&E×100. (C) H&E×400. (D) CD31×100. (E) AE1/AE3×100. (F) p63×100. (G) Ki67×100.

### 3.2 Clinical Follow‐Up

Hematuria temporarily resolved after TURBT but subsequently recurred. Hemoglobin remained stable at 7.4–7.8 g/dL during postoperative days 7–10 but declined to 5.7 g/dL on hospital day 12. The patient and her family declined further transfusions and interventions because of religious beliefs. Best supportive care was initiated, and she was transferred to another hospital for hospice care, where she died 1 week later.

## 4 Discussion

Angiosarcoma of the urinary bladder is an exceptionally rare neoplasm, with only a limited number of cases reported in the literature (Table 1). In most reported cases, treatment involved cystectomy, chemotherapy, or a combination of both, and the natural course of the disease in the absence of treatment has not been clearly characterized in the literature. Reviews of previously reported cases indicate no clear sex predilection, and the median age at diagnosis is 63.5 years (range, 20–89 years) [13, 15]. Reported risk factors include prior pelvic irradiation for prostate or gynecologic malignancies, occupational exposure to vinyl chloride, and tobacco use [4]. Prognostically, the presence of metastatic disease and female sex have been associated with worse outcomes, whereas the use of chemotherapy has been linked to improved survival [16].

Hematuria is the most common presenting symptom of bladder angiosarcoma, reported in approximately three‐quarters of cases [17]. Consistent with these findings, our patient presented with severe gross hematuria resulting in symptomatic anemia that required transurethral electrocoagulation and blood transfusion. Hemostasis of the tumor resection bed was challenging to achieve during the procedure because of the highly vascular nature. Notably, only one previously reported case has specifically described transfusion‐dependent hematuria, suggesting that severe, transfusion‐requiring bleeding may be an uncommon presentation of bladder angiosarcoma [14].

Histologically, bladder angiosarcoma most frequently demonstrates epithelioid morphology and typically expresses endothelial markers, particularly CD31 [13]. In this case, marked nuclear atypia, prominent nucleoli, and a high proliferative index supported a malignant neoplasm. Although CD31 positivity supported endothelial differentiation, the absence of ERG staining and limited immunohistochemical panel represent diagnostic limitations.

With respect to treatment, chemotherapy has been utilized in approximately one third of reported cases, whereas radiotherapy has been applied less frequently [17]. No bladder‐specific chemotherapy regimen exists for angiosarcoma given its extreme rarity, so treatment is extrapolated from general angiosarcoma systemic therapy data, combined with radical cystectomy as the mainstay of local control [18]. The NCCN Soft Tissue Sarcoma guidelines recommend paclitaxel as a preferred systemic therapy option for angiosarcoma, along with anthracycline‐ or gemcitabine‐based regimens [19]. In our case, the patient and her family declined further oncological treatment, including chemotherapy and radiotherapy, and elected best supportive care.

Respecting the patient's religious beliefs was an important aspect of management. Despite receiving explanations regarding her diagnosis and available treatment options, she declined further interventions, including blood transfusions, in accordance with her beliefs. Her decision was supported by her family and the multidisciplinary care team, highlighting the importance of respecting patient autonomy and values when managing aggressive malignancies.

## 5 Conclusion

We report a case of bladder angiosarcoma, a rare, aggressive neoplasm with poor prognosis. Initial symptoms often include gross hematuria. Although prior radiation exposure is a known risk factor, it was absent in this case.

## Ethics Statement

The authors have nothing to report.

## Consent

Informed consent for publication was obtained from the patient.

## Conflicts of Interest

The authors declare no conflicts of interest.

## Data Availability

The data that support the findings of this study are available on request from the corresponding author. The data are not publicly available due to privacy or ethical restrictions.
